# Preclinical Experiments for Hypospadias Surgery: Systematic Review and Quality Assessment

**DOI:** 10.3389/fped.2021.718647

**Published:** 2021-08-09

**Authors:** Tariq O. Abbas, Abubakr Elawad, Aamir Kareem, Abdul Kareem Pullattayil S, Mansour Ali, Abdulla Alnaimi

**Affiliations:** ^1^Regenerative Medicine Research Group, Department of Health Science and Technology, Aalborg University, Aalborg, Denmark; ^2^Pediatric Urology Section, Sidra Medicine, Doha, Qatar; ^3^College of Medicine, Qatar University, Doha, Qatar; ^4^Weill Cornell Medicine Qatar, Doha, Qatar; ^5^Clinical Library, Sidra Medicine, Doha, Qatar; ^6^Urology Department, Hamad Medical Corporation, Doha, Qatar

**Keywords:** hypospadias, animal experiments, quality assessment, clinical translation challenge, translational research

## Abstract

**Background:** There is a steadily growing number of different reconstructive surgical procedures for hypospadias that were tested on animal models prior to their human application. However, the clinical translatability and reproducibility of the results encountered in preclinical urethral reconstruction experiments is considered poor, with significant factors contributing to the poor design and reporting of animal experiments. Our objective was to evaluate the quality of the design and reporting in published articles of urethral reconstructive preclinical studies.

**Methods:** Both PubMed and EMBASE databases were searched for animal urethral repair experiments between January 2014 and September 2019. Internal quality (bias) was evaluated through several signaling questions arising from the Systematic Review Centre for Laboratory animal Experimentation (SYRCLE), while the quality of reporting was assessed by the Animal Research: Reporting of *In vivo* Experiments (ARRIVE) guidelines by scoring of a 20-item checklist.

**Results:** A total of 638 articles were initially screened after the literature search. Employing the inclusion and exclusion criteria, 30 studies were chosen for full-text screening and 21 studies were considered eligible for the quality assessment. The mean score of the checklist was 66%. The elements that accomplished the highest grades included the number of animals utilized, the number in each investigational and control group, and the delineation of investigational conclusions. The items that were least commonly stated comprised information about the experimental method, housing and husbandry, rationalization of the number of animals, and reporting of adverse events. No paper stated the sample size estimation.

**Conclusion:** We found that several critical experiment design principles were poorly reported, which hinders a rigorous appraisal of the scientific quality and reproducibility of the experiments. A comprehensive implementation of the ARRIVE guidelines in animal studies exploring urethral repair is necessary to facilitate the effective translation of preclinical research findings into clinical therapies.

## Introduction

Hypospadias is considered a common birth defect with an incidence of about 1 in 300 live births and has significant clinical and social impacts (1). Furthermore, the reconstructive urethroplasty operations are technically demanding and associated with significant complication rates (1–5). It is considered vitally important that preclinical experiments evaluating the different surgical procedures utilized are well-designed and appropriately reported in order to achieve sound translation to human and generalizability scores (6–9).

Several animal models have been utilized to evaluate several hypospadias repair techniques, with rabbits being the most frequently used (8, 10, 11). This might be because the rabbit's urethra is easily accessed and displays significant functional and structural similarities to human urethra, where a robust envelop of well-vascularized spongiosa encircles a thin epithelial layer underneath (12–16). Furthermore, transurethral endoscopes can be readily used as the size of an adult rabbit's urethra is comparable to that of an infant boy, where most of the hypospadias surgery are mostly conducted.

Several reports have raised concerns that the translation of preclinical experiments to humans has several challenges, including the variations of species and strains with subsequent physiological impact (17), absence of blinding (18), insufficient reporting of technical details, and under-reporting of complications or uncertain results, which could prime false conclusions (19). As a result, in 2010, the Animal Research: Reporting *In vivo* Experiments (ARRIVE) guidelines (20–22) were introduced by the National Centre for Replacement, Reduction, and Refinement (NC3Rs). Despite the increasing utilization of these guidelines, several research territories still struggle to capture the targeted levels of adoption and compliance (23–25). On the other hand, poor study design and incomplete reporting of outcomes might partly explain the hindrance of the clinical translation of urethroplasty procedures (26).

The utilization of systematic reviews to reflect and summarize the findings of animal experimental studies is less common than in clinical studies. Some systematic review features of animal studies need to be tailored accordingly and are mainly affected by bias. Therefore, the degree of translatability of such systematic reviews to clinical practice depends on a sound methodology and the design quality of the included experiments (27). The main goal of this systematic review was to explore these research demands by executing a quality evaluation using the ARRIVE and the Systematic Review Centre for Laboratory Animal Experimentation (SYRCLE) (28) recommendations as checklists. The range of the review has not been restricted to experiments using a certain animal model.

## Methodology

### Literature Search

**Figure 1:**
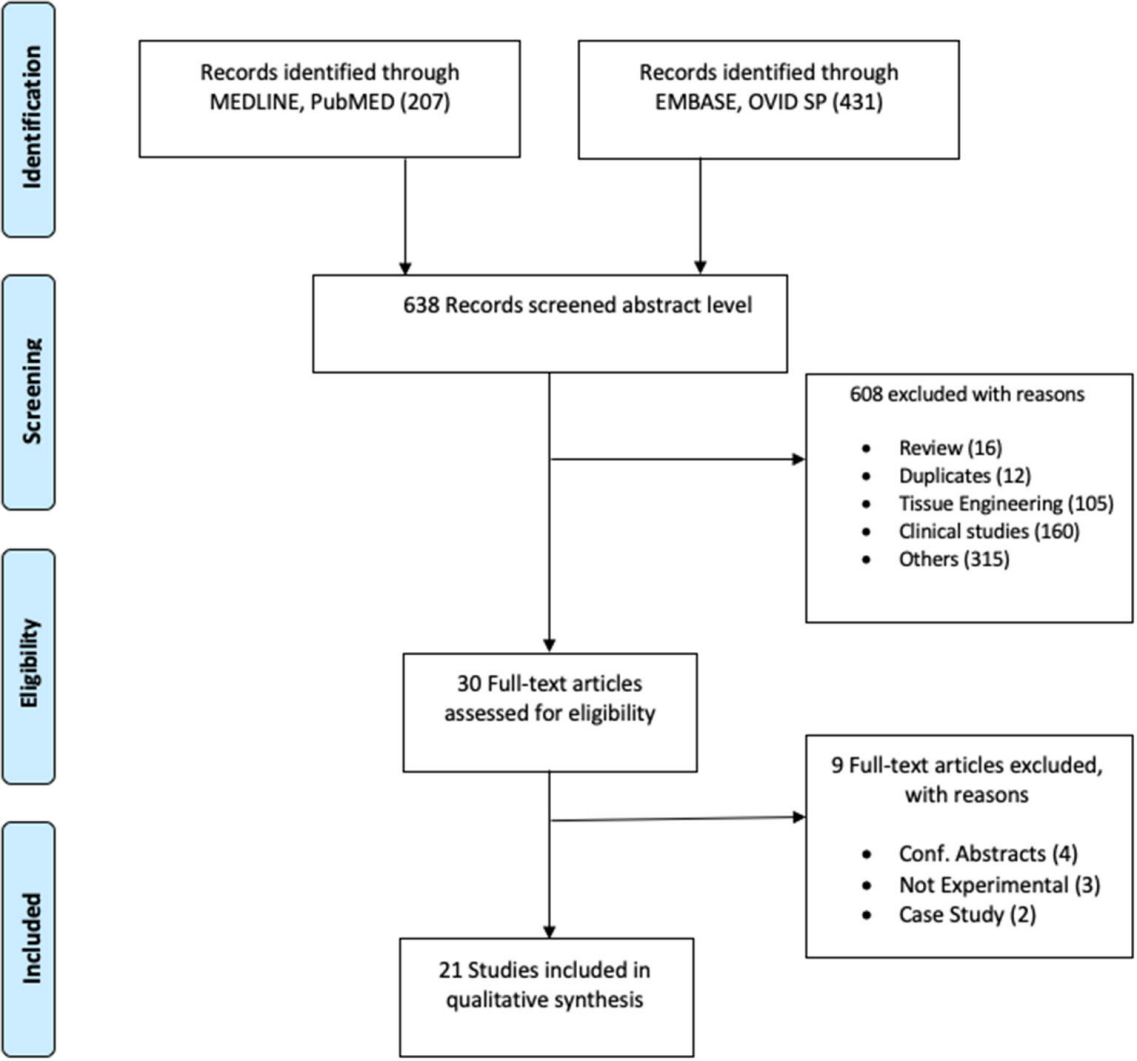
Flowchart of the articles distinguished, included, and excluded.

A search in MEDLINE of the PubMed database and EMBASE of the OVID SP database was conducted in September 2020. The search terms nominated were: urethra, urethroplasty, urethral reconstruction, urethral graft, and animal experimentation reconstructive surgery. The search arenas were organized by database grounds like MeSH term, Text Word, and All Fields suitable to the databases. “Publication date: 01/01/2014 to present” and “English language” filters have been used. Details of the search are represented in the PRISMA flow diagram (Figure 1).

### Screening

All retrieved publications were screened in the abstract level initially by two authors (TA and AKPS). Articles have been excluded, including 12 duplicates (see exclusion criteria in the flowchart in Figure 1). Group discussions resolved disputes regarding the appropriateness of an article. Eligible articles were included for full-text analysis. The reasons for the further exclusion of articles are mentioned in the flowchart.

### Data Extraction

Extraction into a standardized data framework derived from the ARRIVE guidelines (22) (Supplementary Table 1) was conducted separately by three reviewers. In certain ARRIVE questions that were considered to be vital for urethroplasty experiments, the option (NA) was removed and the two options (yes) and (no) were kept for the reviewers to select from. For the possibility of discrepancies between the reviewers, a training phase through detailed descriptions and examples of scoring was conducted with the three reviewers before the commencement of the data collection.

#### Evaluation of the Studies Using the ARRIVE Tool

Information on the ARRIVE guidelines consists of 38 items (Supplementary Table 1). Every item was evaluated as “yes” if the item was reported in the study, “no” if the item was not reported, and “not applicable” if the item was not relevant. All authors read the selected full-text articles independently and extracted the data blinded for the analysis from the other reviewers. Inconsistent data were consequently settled by decision of the third reviewer.

#### Evaluation of the Internal Quality of the Studies

To evaluate the risk of bias of the studies, we used the SYRCLE (28) *via* its 10 signaling questions (Supplementary Table 2). These entries are related to six types of bias: selection bias, performance bias, detection bias, attrition bias, reporting bias, and other biases. If the criteria recommended fitted, we indicated the answer YES (i.e., the risk of bias was low). In contrast, if the guidelines were not met, we assigned the answer NO, symbolizing a considerable risk of bias. If there were inadequate data or the study did not address the expected criteria, either YES or NO could be selected. Alternatively, UNCLEAR was granted (i.e., the risk of bias was unknown). Two independent reviewers did the evaluations, and discrepancies were settled by consensus-oriented discussion.

### Data Analysis

The data were compiled employing a Microsoft Excel spreadsheet and analyzed using the IBM SPSS Statistics, version 21 (IBM Corp., Armonk, NY, USA). For each of the selected studies, a score was calculated, which represents the percentage of positively reported items. The score was calculated using the following formula:

$$\begin{array}{l} Score=\left(\frac{Nyes}{38-Nna}\right)\times 100 \end{array}$$

where *N*_yes_ is the number of “yes” entries, *N*_na_ is the number of “not applicable” entries, and 38 is the total number of items in the ARRIVE guideline. To compare the different scores of the included articles over the years they were published, the Mann–Kendall test was utilized. Statistical significance was set at *P* < 0.05.

## Results

As shown in Figure 1, a total of 638 articles were initially screened after the literature search. Following the inclusion and exclusion criteria, 30 studies were chosen for full-text screening and 21 studies were considered eligible for quality assessment. All studies used male rabbits, except two studies that included Wistar and Sprague–Dawley rats. The most commonly studied approach was evaluating the tabularized incised posterior urethral plate (TIP). These studies comprise a range of approaches for urethral repair, which are summarized in Table 1. The table provides details about the strain, sex, age, weight, number of animals, procedural approach, the complications encountered, and duration of follow-up. The average number of animals in each experiment was 22 and varied between 8 and 38. The average of the post-surgical follow-up duration was 12 weeks and ranged between 2 and 52 weeks (Table 1).

**Table 1:** Overview of the studies included with a summary of the age, sex, and weight of the animal models utilized and evaluation of the hypospadias simulation model and repair.

	**Reference**	**Species**	**Sex**	**Age (weeks)**	**Weight (kg)**	***N***	**Simulation model**	**Repair/technique**	**Details of the outcomes**	**Follow-up duration (weeks)**
1.	(29)	NZW	M	NA	2–2.5	8	Ventral lengthening procedure	Buccal mucosa graft over tunica vaginalis flap for reconstruction of the corpora after corporotomy	• Fistula: 75% • Urinary retention: 50% • Stenosis: 50% • Death; *n* = 2	2, 4, 8, and 12
2.	(30)	Wistar rats	M	NA	0.250	26	Distal hypsopadias	Autologous oral mucosa grafting	• Fistula: *n* = 1 • Infection and graft loss: *n* = 1 • Efficient transdifferentiation process of the grafted oral mucosa	24
3.	(31)	NZW	M	NA	3–3.5	15	Proximal hypospadias	Ventral onlay urethroplasty using an autologous saphenous vein graft	No complications	12
4.	(32)	NZW	M	10	2.5–3	28	Mid-penile hypsopadias	• Group 1: amniotic membrane graft • Group 2: buccal mucosa • Group 3: combined amniotic membrane + buccal mucosa • Group 4: sham (four rabbits died from gastroenteritis)	• Group 1: • Dehiscence (*n* = 1) • Fistula (*n* = 1) • Group 2: • Fistulas (*n* = 2) • Group 3: no complications • Group 4: not reported	8
5.	(33)	NZW	M	NA	4–4.5	9	Proximal hypospadias	Neo- urethro-cutaneostomy	No complications	4
6.	(34)	NZW	M	9.5	~2	38	• Group1: mid-penile hypsopadias (partial circumference) • Group 2: mid-penile hypsopadias (full circumference) • Group 3: sham • Group 4: control	• Group1: TIP • Group 2: mobilization and advancement procedure • Group 3: not reported • Group 4: not reported	• The sham group had the highest stiffness values among all groups in both the dorsal and ventral urethra. • Four rabbits in group 2 were lost due to anesthesia complications (*n* = 3) and suspected infection (*n* = 1)	23
7.	(35)	Sprague–Dawley	M	NA	0.280–0.320	30	Proximal hypospadias	The layers were closed sequentially from the urethra to the skin by a different suture material 6/0.• Chromic catgut • Polyglactic acid • Polydioxanone • Polyglactin 910 • Poliglecaprone 25	The poliglecaprone 25 and PDS groups showed better results regarding urethral lumen volume and the volume of the urethral epithelium	3
8.	(36)	NZW	M	NA	3-4	24	Proximal hypospadias	• TIPU • Perimeatal-based flap urethroplasty (Mathieu) • Onlay island flap urethroplasty	Scarring with TIPU was less apparent than the two other groups.	1, 2, 6, and 12
9.	(37)	NZW	M	9	~2	38	• Group 1: mid-penile hypsopadias (partial circumference) • Group 2: mid-penile hypsopadias (full circumference) • Group 3: sham • Group 4: control	• Group 1: TIP • Group 2: mobilization and advancement procedure • Group 3: not reported • Group 4: not reported	• Four rabbits in group 2 were lost due to anesthesia complications (*n* = 3) and suspected infection (*n* = 1) • The urethral cross-sectional area was significantly larger in the mobilization and advancement group compared to the tabularized incised plate group, shams and controls at the distal distention site, and other groups at the intermediate distention site. • The strain–tension curves were not significantly different between the groups.	23
10.	(38)	NZW	M	NA	2–2.5	16	Mid-penile hypsopadias	• Group 1: outer preputial skin flap • Group 2: inner preputial skin flap	No statistical influence of the flap type on the mean epithelial thickness.	2, 4, 8, and 12
11.	(39)	NZW	M	NA	3–3.5	25	Mid-penile hypsopadias	• Group 1: normal (controls) • Group 2: segmental TIP (single-layer continuous) • Group 3: TIP + mucosal preputial inlay graft	• Same amount of elastic fibers in both groups • Fibrosis occurred in tubularized incised plate urethroplasty with inlay preputial graft.	6
12.	(40)	Sprague–Dawley rats	F	Adult	0.2–0.25	15	Preputial wound	• Group 1 (control): no flutamide (surgically induced hypospadias) • Group 2: congenitally induced hypospadias Mode: received flutamide to establish a rat model of hypospadias	Preputial wound healing was inhibited in rats with hypospadias induced by flutamide	0.5, 1, and 2
13.	(41)	*Oryctolagus cuniculus* rabbits	M	NA	1.6–25	15	Proximal hypospadias	Single- vs. double-layer urethroplasty	Urethral plate repair by the single-layer suturing method could be accompanied by higher epithelialization and wider lumen.	2
14.	(42)	NZW	M	8	2–2.5	16	Proximal hypospadias	Fenestrated buccal mucosa graft	The buccal mucosa fenestrated graft showed complete uptake with keratinization squamous metaplasia and mucosal proliferation of the fenestrated areas.	2, 4, 8, and 12
15.	(43)	NZW	M	25	3.9–4.4	12	Mid-penile hypospadias	Perimeatal flap coverage	• There were no cases of fistula formation. • Meatal stenosis (*n* = 1) • All cases had a satisfactory cosmetic appearance and excellent functional results.	4
16.	(44)	White male rabbits	M	NA	2.5–3.0	35	Proximal hypospadias	The urethral defect was repaired by the everted saphenous vein graft in an onlay fashion.	• The urethra lumen was intact. • No urethral fistula. • No stenosis.	1, 2, 4, 12, and 52
17.	(45)	NZW	M	24	3.8–4.2	28	Mid-penile hypospadias	• Group 0: control (simple closure of urethral defect) • Group A: free penile skin graft • Group B: buccal mucosal graft • Group C: bladder mucosal graft • Group D: pedicle penile skin flap	• Group A: fistula (*n* = 1) • Group D: animal had superficial penile skin loss (*n* = 1) • The urethrograms confirmed the maintenance of a normal-caliber urethra.	12
18.	(46)	NZW	M	Adult	3–3.5	27	Mid-penile hypsopadias	• Group 1: control, non-operated • Group 2: TIP urethroplasty • Group 3: TIPG	• The elasticity of the TIP or TIPG neourethra tended to be reduced when compared to controls. • The placement of an inlay graft on the dorsal incised area did not increase the compliance.	6
19.	(47)	NZW	M	NA	NA	16	• Group 1: longitudinal dorsal penile urethrotomy • Group 2: distal hypospadias	Foreskin flap urethroplasty onlay to the albuginea	• Group 1: less inflammatory process • Group 2: two animals have both the fistula and stricture and one animal has only the fistula. Fibrosis was slightly more intense.	2, 4, 8, and 12
20.	(11)	NZW	M	8	2.5	16	Mid-penile hyposopadias	Bracka's urethroplasty	No complications	2, 4, 8, and 12
21.	(48)	NZW	M	NA	3–4	12	Mid-penile hyposopadias	Dorsal inlay graft urethroplasty	Regardless of incision depth at TIPU, the average gain in urethral width was only 2 mm.	2 and 4

*M, male; NA, not available; PDS, polydioxanone; TIP, tabularized incised posterior urethral plate; TIPU, tabularized incised plate urethroplasty; TIPG, TIP + inner preputial graft*.

### Results of the External Quality (Reporting) Assessment

**Figure 2:**
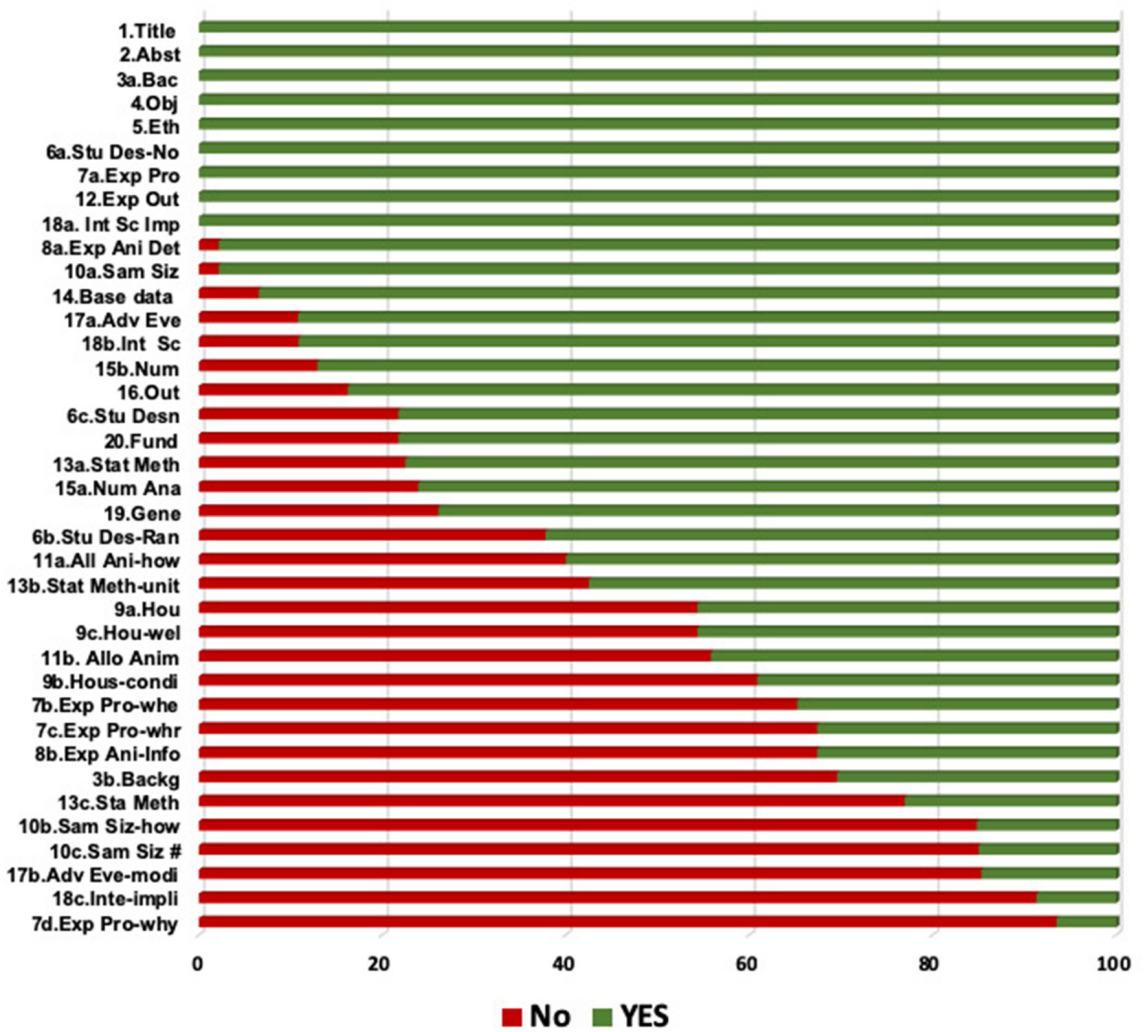
Bar chart displaying the frequencies of the options “Yes” and “No” among the 22 articles selected for the analysis. Items are indicated by their corresponding numbers and a label associated with their content. *Alloc Ani*, allocation of animals; *Exp ani*, experimental animals; *Basel*, baseline data; *Fund*, funding; *Generali*, generalizability; *Stu desig*, study design; *Sample*, sample size.

**Figure 3:**
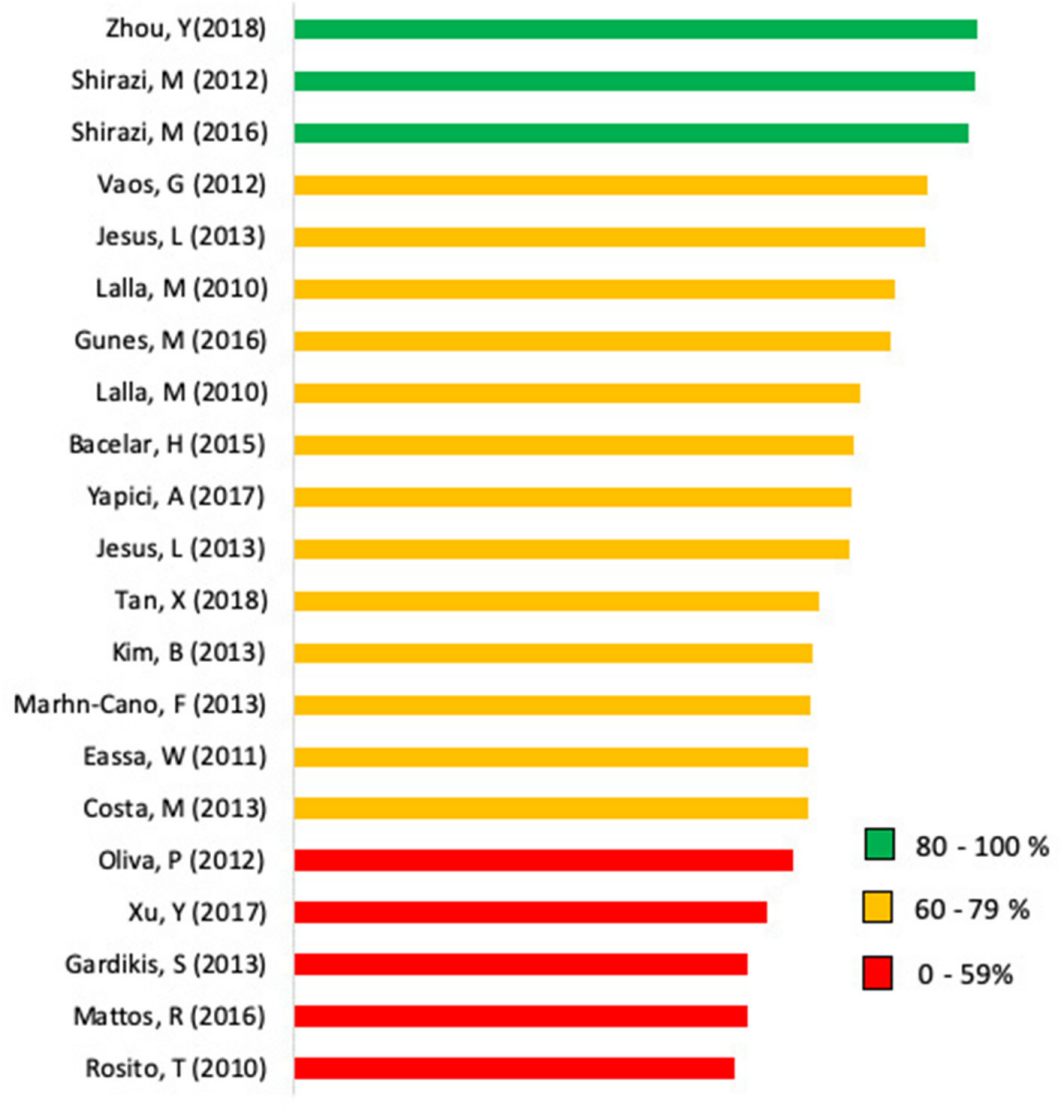
Bar chart displaying the scores of the analyzed articles. Scores represent the percentage adherence to the ARRIVE (Animal Research: Reporting of *In vivo* Experiments) guidelines on a scale from 0 to 100.

The frequencies of the options “yes,” “no,” and “n/a” of the selected studies according to the ARRIVE checklist are shown in Figure 2. Calculation of the scores of each individual study is presented in Figure 3. The mean checklist score of the studies published from 2014 to 2019 was 66%.

The background of the studies was described adequately, including the rationale and the context in all the experiments. The elements that accomplished the highest grades comprised the number of animals utilized, the number in each investigational and control group, and the delineation of investigational conclusions. The items that were least commonly stated comprised information about the experimental method, housing and husbandry, rationalization of the number of animals, and reporting of adverse events.

No tendency or steady pattern in the grade of the scores could be recognized over the studied duration (2014–2019) as the Kendall's rank correlation coefficient (tau) was unfolded to be very low at 0.055 (*P* = 0.701).

**Figure 4:**
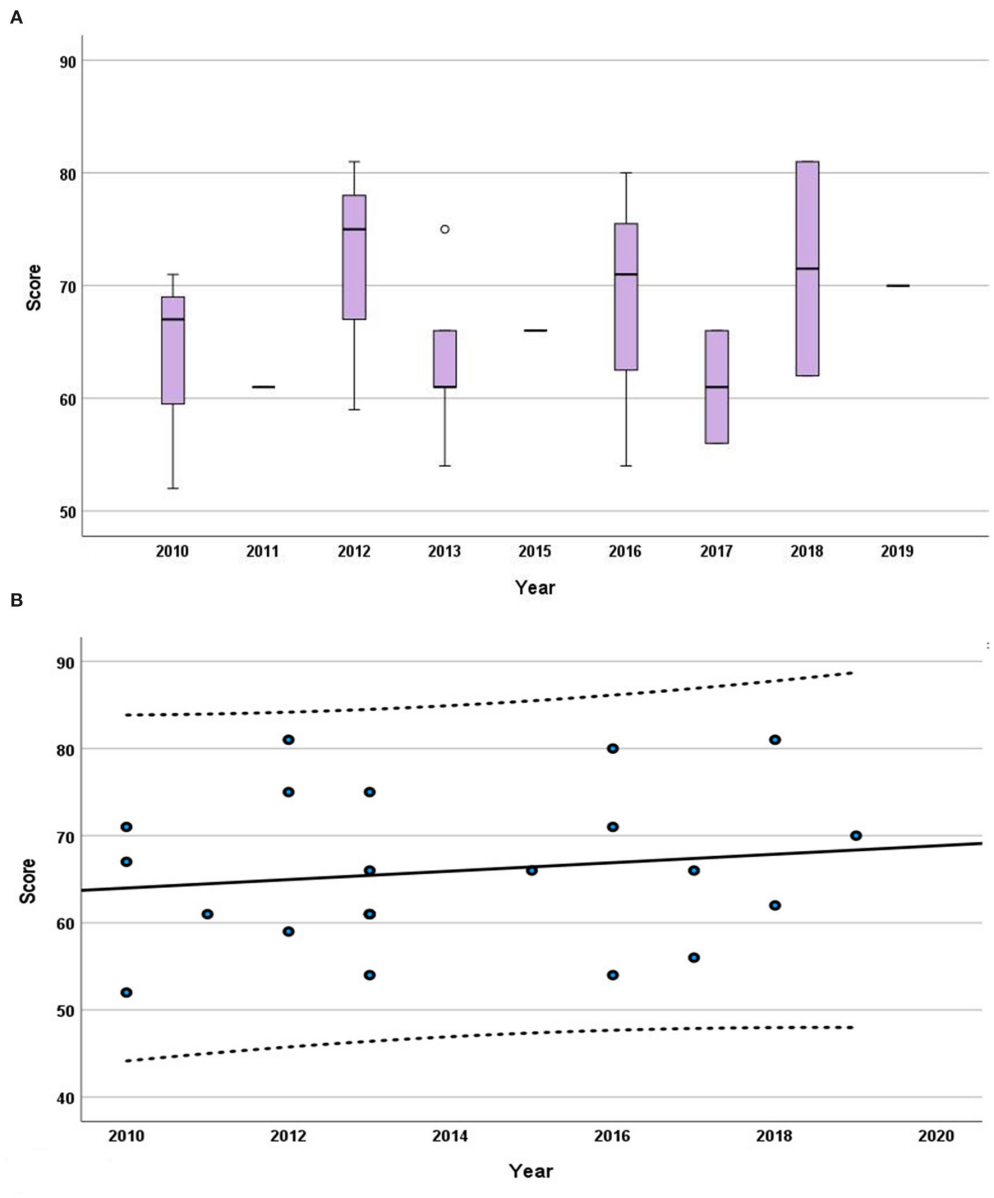
Progression of the grades throughout the years. **(A)** Box plot displaying the median, interquartile range, minimum, and maximum of the scores for each year from 2014 to 2019. **(B)** The trendline presented is from the Mann–Kendall trend analysis on the median of the scores. The *interrupted lines* indicate the 95% confidence interval.

Regarding the study design, numerous vital elements were poorly communicated. Only one study answered the item (7d) about study processes. Recording of randomization scored 62%. None of the 21 studies reported sample size estimation. The least frequently reported items (reported in ≤ 20% of the studies) were items 18c (interpretation), 10b and 10c (sample size), 7d (experimental procedures), and 17b (adverse events). As is evident from Figures 3, 4, none of the analyzed studies fully complied with the ARRIVE guidelines.

### Results of the Internal Quality (Bias) Assessment

**Figure 5:**
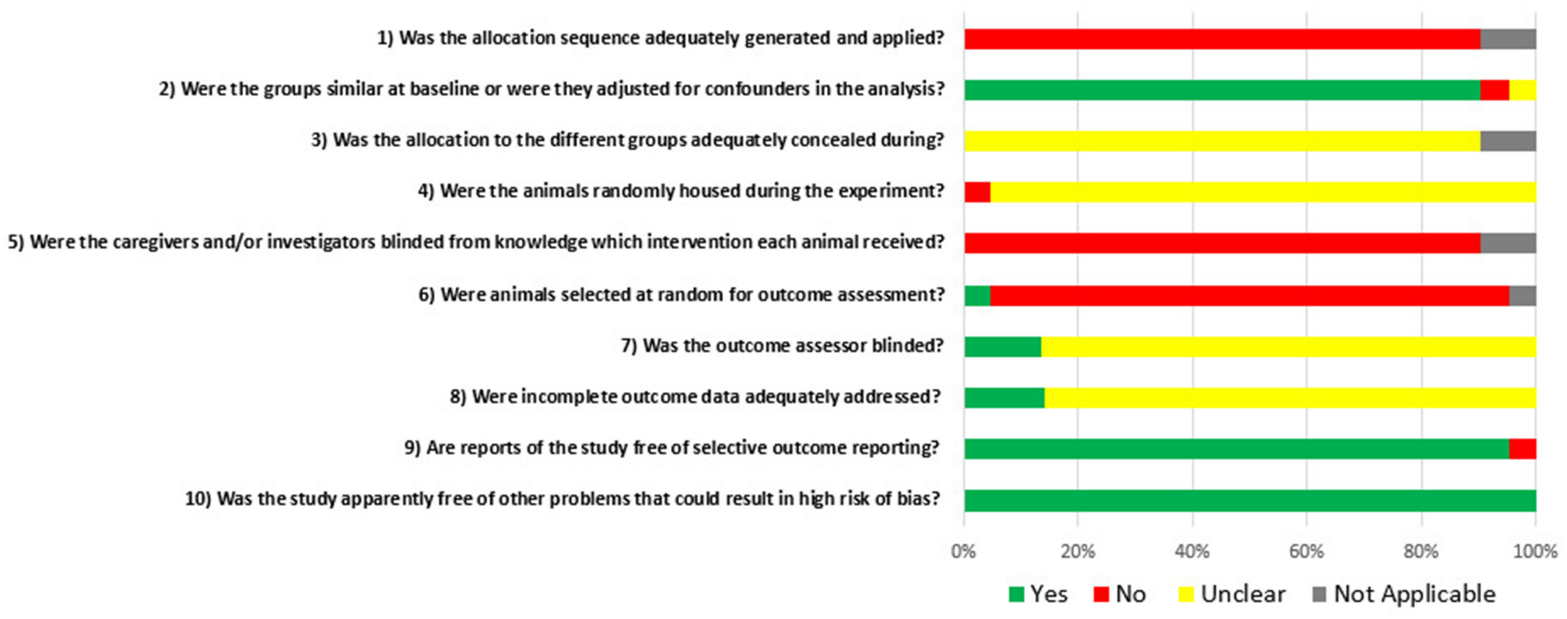
Risk of bias, averaged per each signal question. Yes = low risk of bias; No = high risk of bias; Unclear = unclear risk of bias; and Not applicable.

Figure 5 displays the global grades of the bias risk appraisal of the 21 studies involved in this systematic review. Of the studies, 92% stated that the experimental groups were similar at baseline or were adjusted for confounders. None of the papers described whether the allocation to the different groups during the randomization process was concealed. Sixteen percent of the studies reported that the outcome assessment has been blinded.

## Discussion

This systematic review unfolded that the reporting quality of some crucial pieces was commonly poor in preclinical studies of hypospadias repair, which did not seem to progress over the years. Preclinical studies play an important role in scientific progress and the discovery of new and potentially successful surgical procedures, provided that they are designed, conducted, assessed, and sufficiently reported according to internationally agreed guidelines. Several principal parts of the study design are frequently missed, which significantly adds to failure of reproducibility following these experiments, and urethral reconstructive studies seem not to be an exception. Fundamental experimental design components are often overlooked in scientific papers, which contributes to the irreproducibility of the experiments (49), and preclinical studies investigating urethral repair do not appear to be an exemption.

This study revealed that there is still room for methodological improvements of experiments on animals in the urethral reconstruction and hypospadiology fields. Most domains were judged to have an unclear risk of bias, and therefore, it is not possible to determine the degree of bias of the described treatment effects. Note that the risk of bias and the quality of reporting should be considered distinct from each other. Although, the former relates to the internal efficacy of a trial, the latter points to how researchers report their conclusions. Although, personal communication with the authors of the study might be an attempt at clarifying dubious or lack of information, this does not warrant the correctness of the information rendered (50).

Strikingly, particulars to safeguard the reproducibility of such experiments, like animal housing, husbandry, and anesthetics, were seldom communicated, which might meaningfully disturb the study conclusions (51). Prager et al. (52) revealed that different animal husbandry conditions could affect several research outcomes. Furthermore, caring for the study animals in tiny cages or in big groups following urethroplasties likely brings hazard of infections, probabilities of dislodgements of stents, and surgical site trauma.

None of the included experiments reported sample size calculation similar to the previous signal that displays scarcity of reporting concerning sample size calculation in animal studies (53). Additionally, not defining the study design before the commencement of experiments could result in inappropriate analysis of the null hypothesis and insufficient sample size, potentially leading to debatable conclusions. The required sample size of equivalence trials is usually larger than that for superiority trials (54). Experiments with low power may create false-negative results, i.e., so-called type II errors (55). The low average number of animals in most studies could be partly rationalized by the high cost and the difficulty of testing, handling, and monitoring animals throughout the experiment. Nonetheless, approaches to verify the number of animals used exist, such as performing previous pilot investigations or utilizing Mead's resource equation in situations where there is no information on the standard deviation and it is challenging to define an effect size (56).

Only a few of the included studies have deliberated the “why and how” regarding the type of animal model employed and its applicability to human pathology. We consider that arguments are of noteworthy significance in all surgical reconstructive experiments because of the substantial variations between the genital anatomy of humans and animals. We found that blinding was not steadily described. However, it might be impractical to blind the investigator performing different urethroplasty procedures. Therefore, we did not attempt to analyze blinding within the included studies.

An implementation approach to increasing compliance of reporting quality would be stringent polices by the editorial committees of the journals (57). However, a recent randomized controlled trial exposed that instructing the accomplishment of an ARRIVE checklist throughout submission of the paper, with no additional pressure on reporting by the editorial team, did not encourage compliance (58). The recently published PREPARE (Planning Research and Experimental Procedures on Animals: Recommendations for Excellence) guidelines (59) can likewise aid in implementing more consideration to investigational precision at an earlier stage of the planning course.

The limitations of this review include the potential subjectivity of the assessment by the evaluators. Yet, the pronounced inter-observer consensus established that the assessors had a rather similar approach to applying these guidelines. Additionally, the rather small number of studies involved restricted a broad appraisal of the conditions persuading reporting. Calculating a summary score for each study using the SYRCLE was not done as a summary score inevitably involves assigning “weights” to specific domains in the tool, and it is difficult to justify the weights assigned. Also, these weights might differ per outcome and review. Using the existing animal experimental literature is also challenging because the current reporting quality of animal studies is low; several details regarding the housing conditions or timing outcome assessment are often unreported. Users also indicated that they had to judge many entries as having an “unclear risk of bias.”

## Conclusion

This review exposed that the experiments testing urethral reconstructive procedures suffer from significant internal (design/bias) limitations and significant reporting shortages. We encourage the usage of the ARRIVE procedures in all animal experiments to benefit the production of manuscripts that deliver defined conclusions of scientific value.

## Data Availability Statement

The original contributions presented in the study are included in the article/Supplementary Material, further inquiries can be directed to the corresponding author/s.

## Author Contributions

TA conceived the article, planned, and prepared its structure. TA performed the bibliographical search along with AP, AE, AK, and AA. AE, TA, AK, and AP analyzed the results. TA wrote the manuscript draft. TA, AK, AP, MA, AA, and AE edited sections of the manuscript and contributed to the critical revision of the final draft.

## Conflict of Interest

AA is employed by Hamad Medical Corporation. The remaining authors declare that the research was conducted in the absence of any commercial or financial relationships that could be construed as a potential conflict of interest.

## Publisher's Note

All claims expressed in this article are solely those of the authors and do not necessarily represent those of their affiliated organizations, or those of the publisher, the editors and the reviewers. Any product that may be evaluated in this article, or claim that may be made by its manufacturer, is not guaranteed or endorsed by the publisher.
