# Supplementary material for: Preclinical Experiments for Hypospadias Surgery: Systematic Review and Quality Assessment
Source: Front Pediatr. 2021 Aug 9;9:718647. doi: 10.3389/fped.2021.718647 (PMC8386350; doi:10.3389/fped.2021.718647)
Supplement: Supplementary file 2 [file Table_2.docx]

**Supplementary material Table 2.**

|  | **Signal Questions** |
| --- | --- |
|  | Was the allocation sequence adequately generated and applied? |
|  | Were the groups similar at baseline or were they adjusted for confounders in the analysis? |
|  | Was the allocation to the different groups adequately concealed during? |
|  | Were the animals randomly housed during the experiment? |
|  | Were the caregivers and/or investigators blinded from knowledge which intervention each animal received? |
|  | Were animals selected at random for outcome assessment? |
|  | Was the outcome assessor blinded? |
|  | Were incomplete outcome data adequately addressed? |
|  | Are reports of the study free of selective outcome reporting? |
|  | Was the study apparently free of other problems that could result in high risk of bias? |
